# Role of KCNMA1 in Breast Cancer

**DOI:** 10.1371/journal.pone.0041664

**Published:** 2012-08-10

**Authors:** Martin Oeggerli, Yuemin Tian, Christian Ruiz, Barbara Wijker, Guido Sauter, Ellen Obermann, Uwe Güth, Inti Zlobec, Matthias Sausbier, Karl Kunzelmann, Lukas Bubendorf

**Affiliations:** 1 Institute of Pathology, University of Basel, Basel, Switzerland; 2 Institute of Physiology, University of Regensburg, Regensburg, Germany; 3 Institute of Pathology, University Medical Center Hamburg-Eppendorf, Hamburg, Germany; 4 Department of Gynecology and Obstetrics, University Hospital Basel, Basel, Switzerland; 5 Institute of Pathology, University of Bern, Bern, Switzerland; 6 Institute of Pharmacy, University of Tübingen, Tübingen, Germany; Virginia Commonwealth University, United States of America

## Abstract

*KCNMA1* encodes the α-subunit of the large conductance, voltage and Ca^2+^-activated (BK) potassium channel and has been reported as a target gene of genomic amplification at 10q22 in prostate cancer. To investigate the prevalence of the amplification in other human cancers, the copy number of *KCNMA1* was analyzed by fluorescence-*in-situ*-hybridization (FISH) in 2,445 tumors across 118 different tumor types. Amplification of *KCNMA1* was restricted to a small but distinct fraction of breast, ovarian and endometrial cancer with the highest prevalence in invasive ductal breast cancers and serous carcinoma of ovary and endometrium (3–7%). We performed an extensive analysis on breast cancer tissue microarrays (TMA) of 1,200 tumors linked to prognosis. *KCNMA1* amplification was significantly associated with high tumor stage, high grade, high tumor cell proliferation, and poor prognosis. Immunofluorescence revealed moderate or strong KCNMA1 protein expression in 8 out of 9 human breast cancers and in the breast cancer cell line MFM223. KCNMA1-function in breast cancer cell lines was confirmed by whole-cell patch clamp recordings and proliferation assays, using siRNA-knockdown, BK channel activators such as 17ß-estradiol and the BK-channel blocker paxilline. Our findings revealed that enhanced expression of KCNMA1 correlates with and contributes to high proliferation rate and malignancy of breast cancer.

## Introduction

BK (KCNMA1) potassium channels are a diverse class of ion channels expressed in many different cell types [1]. They modulate numerous physiological processes in excitable and non-excitable tissues and take part in forming macromolecular signaling complexes [2], [3]. The protein encoded by KCNMA1 represents the pore-forming α subunit of the α-subunit of the large conductance, voltage and Ca^2+^-activated (BK) K^+^ channel. The α-subunit can form macromolecular complexes with four different types of auxillary β-subunits and local Ca^2+^ influx channels [4]. Because of the large number of protein interactions and activating factors influencing BK channel function, including intracellular Ca^2+^, membrane voltage, pH, shear stress, carbon monoxide, phosphorylation states, as well as G-proteins and steroid hormones, it is generally difficult to predict the role of BK channels in a given tissue [5]. Moreover, phosphatidylinositol 4,5-bisphosphate (PIP_2_), PI3K and PTEN can regulate BK channel activity [6], [7]. Finally, BK channel function and pharmacological properties are fine-tuned by differential splicing and depend on the presence of auxiliary β-subunits (β1–β4) [8].

In many diseases, defective regulation/or expression of BK channels have repeatedly been associated with altered cell cycle progression [9], cell proliferation [10], [11], [12], [13], [14], [15], [16], and cell migration [17], [18]. These factors are fundamental to the development of cancer [19], [20]. As demonstrated in electrophysiological studies on cervical and breast cancer cells, BK channels are directly activated by estrogens, which have an essential role in cancers of the uterus, breast and prostate [21], [22]. Specific blockade of BK channels leads to membrane depolarization, cell cycle progression and inhibition of cell proliferation [1], [23]. Early reports pointed to high levels of KCNMA1-expression in human glioblastomas [24]. A number of subsequent reports pointed to a more general role of BK channels in different types of cancer, although this not seems to apply to all (Cambien et al., 2008). We previously detected genomic amplification of the BK channel encoding gene *KCNMA1* in 16% of late-stage prostate cancers, identifying *KCNMA1* as one of the most common amplifications in prostate cancer [13]. We found that knockdown of *KCNMA1* by siRNA and specific BK channel blockade by iberiotoxin inhibited cell proliferation of the prostate cancer cell line PC3, which carries an amplification of *KCNMA1*. This study suggested a specific role of KCNMA1 in the transition from hormone-sensitive to hormone-insensitive and castration-refractory prostate cancer [13].

The aim of this study was to investigate the prevalence of *KCNMA1* amplification in cancers beyond prostate cancer, and to explore its functional and prognostic consequences.

## Methods

### Fluorescence *in situ* hybridization (FISH), probes, and clinical specimens

The bacterial artificial chromosome (BAC) RP11-428p16, which contains major parts of the *KCNMA1* gene sequence and CEP 10 (Spectrum orange labelled; Vysis), were utilized as described previously (Bloch *et al.*, 2006). The following criteria were applied for amplified FISH-amplified results: *KCNMA1*-probe/centromer 10-signal ratio of at least 2, and at least 5 signals of *KCNMA1*-probe.

### Tissue Microarrays (TMA)

Formalin-fixed and paraffin-embedded clinical specimens from the archive of the Institute of Pathology, University Hospital Basel, Switzerland, were used for the construction of all TMAs [25]. The breast cancer TMA was constructed from a total of 2'222 formalin-fixed, paraffin-embedded tumours, as previously described [26]. The median patient age was 62 (range, 26–101) years and the mean follow-up time was 68 months (range, 1–176). The information on the clinical follow-up were retrieved from the patient charts. Tumour data regarding histologic subtype, TNM classification, Bloom-Richardson- Elston-Ellis (BRE) grade, and diameter were obtained from pathology reports. Additionally, frozen breast cancer specimens were used for immunofluorescence. This study was approved by the ethical committee Basel (EK 142/09). According to the decision of the ethical committee a written informed consent by the patients was not needed due to the retrospective nature of the study.

### Cell lines and cell culture

T47D [27], SKBR3 [28] and PC3 [13] cell lines were obtained from American Type Culture Collection (ATCC, LGC Promochem, Molsheim Cedex, France) and grown under standard cell culturing conditions in Optimem cell culture medium (Invitrogen, Carlsbad, CA), supplemented with 1% penicillin/streptomycin (Amimed, Basel, Switzerland) and 10% FCS (Amimed) at 37°C/5% CO_2_. Trypsin-EDTA (Amimed) was used as a transferring reagent. Cell lines MFM223 [29], MCF7 [30], LNCaP [13] and BPH1 [13] were obtained from German Collection of Microorganisms and Cell Cultures (DSMZ, Braunschweig, Germany) and grown under the same conditions. In case of MCF7, the Optimem medium was replaced by RPMI 1640-cell culture medium. PC346 [31] and PC3456C [31] were obtained from G. Jenster (Department of Urology, Josephine Nefkens Institute, Erasmus Medical Center, P.O. Box 1738, 3000 DR, Rotterdam, The Netherlands) and grown under standard conditions.

### RNAi and proliferation assays

Transfection was performed following the small-interferent RNA (siRNA) sequences transfection protocol for Lipofectamine 2000 (Qiagen, USA). 4 hours after transfection cell culture medium was replaced and supplemented with 10% FCS, to trigger normal cell growth. Nonsense RNAi (nsRNA) was used as a negative control for KCNMA1 siRNA. Relative levels of KCNMA1 expression were determined in replicate cultures by real-time PCR. For proliferation assays replicate cultures were grown in parallel, to allow repeated cell harvesting and counting. Cell counting was performed, using a ‘CASY’ cell counter (Innovatis, Reutlingen, Germany) and standard counting procedures were followed to determine cell quantity. The efficiency of the RNAi on KCNMA1 knock-down was controlled in replicate cultures that were grown in parallel.

### Immunofluorescence of frozen tissue samples, cytospins and microscopy

Trypsinized cells were harvested and washed in PBS twice. Aliquots of 100 µL were centrifuged for 2 minutes and dried with a ventilator. Frozen tissue samples and cytospins were incubated with an anti-KCNMA1 polyclonal antibody and prepared as previously described [13]. Images were obtained using a Zeiss Axioplan 2 fluorescence microscope (Zeiss, Jena, Germany), equipped with an appropriate filter set. The selection of target cells was based on clear visibility in DAPI-staining. An ISIS-digital camera (MetaSystems, Altlussheim, Germany) was set up to capture multi-stack images. The resultant images were resized, cropped and had their levels and contrast adjusted accordingly. PBS-washed slides were used as negative controls.

### Immunohistochemistry

Standard indirect immunoperoxidase procedures were used for the detection of Ki67 (clone MIB1, prediluted, DAKO, Glostrup, Denmark), estrogen receptor-α (ER-α clone 6F11, dilution 1∶1'000, Novocastra Laboratories, Newcastle, UK) and androgen receptor (AR clone 156C135.1, dilution 1∶50, Imgenex Corp., San Diego, USA) in paraffin-embedded PCA tissue sections by standard protocols, as previously described [32]. Ki67 LI was defined as the fraction of tumour cells showing any nuclear Ki67 immunoreactivity. 100 tumour cells were counted per tissue spot to determine the Ki67 LI. Analysis was performed using Bond Max automated immunohistochemistry slide staining system (Leica Microsystems GmbH, Germany).

### RNA extraction and cDNA synthesis, quantitative RT-PCR

Total RNA from cell lines was extracted using RNeasy Mini Kit (QIAGEN, Hilden, Germany) according to the instructions of the manufacturer. RNA concentrations were determined with Nanodrop spectrophotometer (Witec AG, Littau, Switzerland). Real-time PCR was performed using the LightCycler system and LightCycler FastStart DNA Master Hybridization Probes kit (Roche, Hilden, Germany). Primers were obtained from TIB MolBiol (Berlin, Germany): KCNMA1-for: TggCCTCCTCCATggTgA, KCNMA1-rev: TTCTgggCCTCCTTCgTCT, GAPDH-for: gAAggTgAAggTCggAgTC, GAPDH-rev: gAAgATggTgATgggATTTC, KCNMA1-FL: AgCgTCCgCCAgAgCAAgAT-FL, KCNMA1-LC: LC640-ATgAAgAggCCCCCgAAgAAAgT-PH, GAPDH-FL: AggggTCATTgATggCAACAATATCCA-FL, GAPDH-LC: LC640-TTTACCAgAgTTAAAAgCAgCCCTggTg-PH. PCR conditions were: Activation 10 min 95°C, annealing 5 s at 54°C, elongation 15 s at 72°C, 40 cycles. Relative levels of expression (normalized against GAPDH) were determined according to [33].

### Patch clamping

MCF-7 and MFM-223 cells grown on glass cover slips were mounted on the stage of an inverted microscope (IM35, Zeiss, Göttingen, Germany), kept at 37°C and continuously perfused with Ringer solution of the following composition (mmol/l): NaCl 145, KH_2_PO_4_ 0.4, K_2_HPO_4_ 1.6, D-glucose 5, MgCl_2_ 1, Ca-gluconate 1.3, pH 7.4). Experiments were performed in the fast whole cell configuration as described previously [13]. In brief, cells were voltage clamped from −60 to +40 mV and patch pipettes had an input resistance of 2–4 MΩ, if filled with a solution containing (mM): KCl 30, K-gluconate 95, NaH_2_PO_4_ 1.2, Na_2_HPO_4_ 4.8, EGTA 1, Ca-gluconate 0.758, MgCl_2_ 1.034, D-glucose 5, ATP 3. The pH was set to 7.2 and Ca^2+^ activity was 0.1 µM. The access conductance was measured continuously, remaining between 60–100 nS. Membrane conductance G_m_ was calculated from the measured current (I) and V_c_ values according to Ohm's law.

### Materials and statistics

17*β*-estradiol (Sigma-Aldrich, Buchs, Switzerland) was dissolved in 100% ethanol to build a stock solution of 0.5 µM, which was further diluted to a final concentration of 10 nM, using the appropriate cell culture medium. Paxilline (Sigma-Aldrich, Buchs, Switzerland) was dissolved in 100% DMSO to build a stock solution of 50 mM, and then further diluted in phenol red-free medium supplemented with 5% dextran-coated charcoal-stripped FCS [27] to a final concentration of 15 µM. Prior to the experiments, standard growth curves were calculated to adjust optimal conditions for all cell lines and reagents, respectively. For all patch clamp experiments, Student's t-test P-values<0.05 were accepted to indicate statistical significance (*).

## Results

### Large-scale FISH analysis on multi-tumour and breast cancer TMA

**Figure 1:**
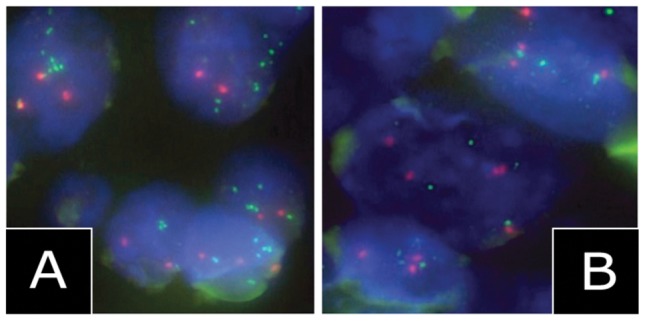
Amplification of KCNMA1 in breast cancer. FISH analysis of *KCNMA1* in amplified (left) and non-amplified (right) breast cancer specimens (magnification ×1000).

Previously, we reported amplification of *KCNMA1* in 16% of advanced-stage human prostate cancers [13]. In the present study, we examined the prevalence of the *KCNMA1* amplification in the whole range of human cancer and analyzed a large multi-tumour TMA, containing 2445 tissue spots from 118 different tumour types by FISH, using a KCNMA1-specific BAC probe (**Table 1**). FISH was successful in 2172 samples (88.8%). Amplification of *KCNMA1* was restricted to 10 of 603 (1.7%) gynecological tumours known to be influenced by estrogens (**Table 1**, **Figure 1**). Using FISH, the results were validated on a large breast cancer TMA (**Table 2**). Amplification of *KCNMA1* was found in 23 of 1200 breast cancers (1.9%) and was significantly associated with high tumour grade (p<0.001), high tumour cell proliferation (p<0.01) and poor tumour-specific survival (Log-Rank p = 0.031).

**Table 1:** Amplification of KCNMA1 in tumours.

Tumor entity	subtype	n	amp	amp (%)
1. Skin		151	0	0
2. Respiratory tract		136	0	0
3. Gynecological tract		599	10	1.7
endometrium	serous carcinoma	30	1	3.3
endometrium	endometrioid carcinoma	46	0	0
endometrium	stromal sarcoma	3	0	0
endometrium	carcinosarcoma	26	0	0
breast	ductal carcinoma	44	3	6.8
breast	medullary carcinoma	43	2	4.7
breast	lobular carcinoma	28	0	0
breast	mucinous carcinoma	34	0	0
breast	tubular carcinoma	26	0	0
breast	cribriform carcinoma	21	0	0
breast	apocrine carcinoma	10	0	0
breast	phyllodes tumor	35	0	0
ovary	malignant müllerian mixed tumor	4	1	25.0
ovary	serous carcinoma	48	3	6.3
ovary	mucinous carcinoma	26	0	0
ovary	Brenner tumor	21	0	0
ovary	endometroid carcinoma	14	0	0
vagina	squamous cell carcinoma	19	0	0
vulva	squamous cell carcinoma	54	0	0
cervix	squamous cell carcinoma	43	0	0
cervix	adenocarcinoma	22	0	0
cervix	adenosquamous carcinoma	2	0	0
4. Gastrointestinal tract		383	0	0
5. Urogenital tract		239	0	0
6. Endocrine organs		161	0	0
7. Haematolymphatic system		116	0	0
8. Brain		171	0	0
9. Soft tissue		216	0	0
**Sum**		**2172**	**10**	**0.5**

FISH analysis in a multi-tumour TMA with 2445 specimens from 118 different tumour types. The amplification was restricted to a small fraction of breast cancer and rare cases of ovarian and endometrial cancer.

**Table 2:** Amplification of KCNMA1 in breast cancer.

Breast cancer subtype	n	amp	amp (%)
papillary	18	1	5.6
cribriform	36	1	2.8
invasive ductal	881	20	2.3
invasive lobular	139	1	0.7
medullary	37	0	0
tubular	35	0	0
mucinous	31	0	0
apocrine	7	0	0
clear-cell	5	0	0
medullary atypical	4	0	0
metaplastic	5	0	0
neuroendocrine	2	0	0
**Sum**	**1200**	**23**	**1.9**

Summary of the results from FISH analysis of KCNMA1-amplification in 1200 breast cancers.

**Figure 2:**
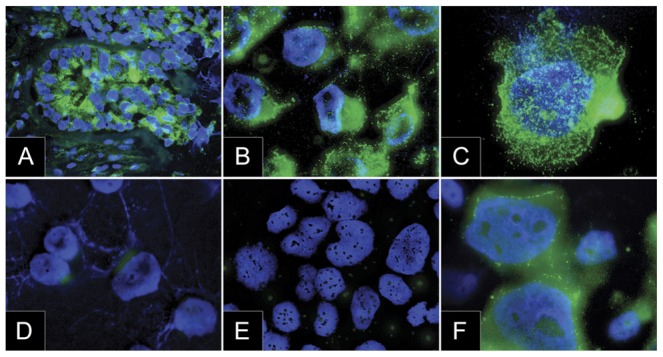
Expression of KCNMA1 (micrographs). Immunofluorescence on a frozen tumour tissue sample and cytospins of different cell lines. Stainings represent BK-specific antibody (green) and DAPI (blue). Positive KCNMA1-staining in a KCNMA1-amplified frozen breast cancer specimen (A), positive staining in KCNMA1 amplified MFM223 at low (B) and high (C) magnification, negative staining in MCF7 (D) and T47D (E) at low magnification, and positive staining of the KCNMA1 amplified prostate cancer cell line PC3 (F; positive control) shown at high magnification. Bar = 10 µm.

**Figure 3:**
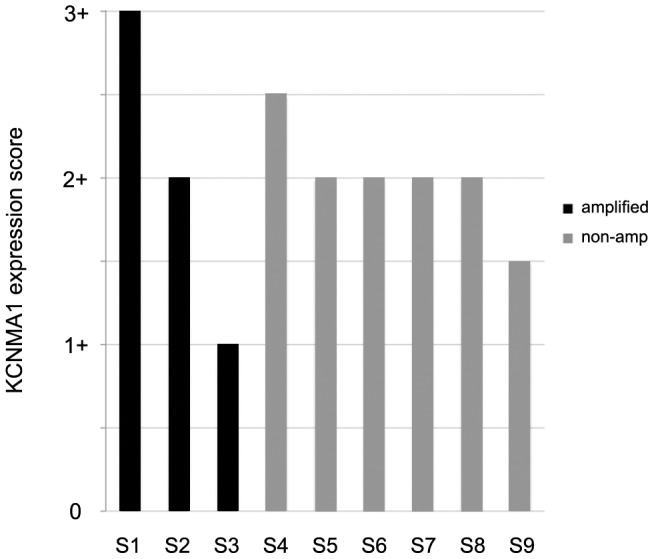
Expression of KCNMA1. Immunofluorescence in amplified (n = 3) and non-amplified (n = 6) breast cancer tissue sections. KCNMA1 expression was scored independently by two persons. Bars represent average scores (scores ranged from: 0, 1, 2, to 3).

Analysis frozen sections from nine human breast cancers by immunofluorescence revealed strong expression of BK channels in seven, and weak expression in two specimens. There was no correlation between amplification of *KCNMA1* and BK expression in these specimens, suggesting that KCNMA1 can also be up regulated by mechanisms other than genomic amplification (**Figure 2**). The *KCNMA1*-amplified breast cancer (MFM223) and prostate cancer (PC3; positive control) cell lines were strongly positive for BK channels, while non-amplified breast cancer cell lines T47D and MCF7 were only weakly positive or negative as evaluated by immunofluorescence (**Figure 3**).

### Association between KCNMA1 amplification and cell proliferation, ER-α and AR expression

**Figure 4:**
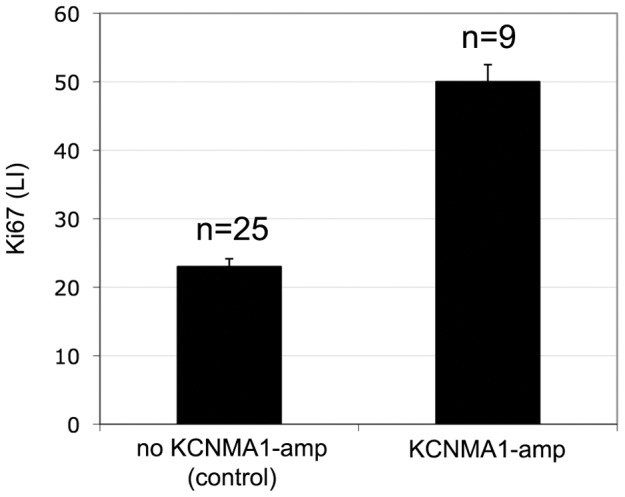
Association of KI67 with KCNMA1. Mean values from MIB1 (Ki67)-staining in 34 breast cancers, which have been analysed by FISH. The results reveal a significant association between KCNMA1 and fast tumour growth in breast cancer (p<0.002).

To explore the association between amplification of *KCNMA1* and tumour cell proliferation, we constructed a TMA with 34 specimens enriched for *KCNMA1* amplified breast cancers (9 with and 25 without *KCNMA1* amplification) using two tissue cores per specimen. The specimens were also analyzed by IHC for expression of ER-α, AR and Ki67 (Mib1). *KCNMA1* amplification was significantly associated with high tumour cell proliferation (defined as Ki67 LI>30%; p<0.002) (**Table 3**, **Figure 4**). Interestingly, a positive AR expression was detected in 14 of 25 (56%) of the non-*KCNMA1* amplified tumours but in only one of nine (11%) *KCNMA1* amplified tumours (p<0.05). Immunohistochemical AR positivity was not associated with proliferation (data not shown). ER-α was not associated with KCNMA1-amplification status of Ki67 (data not shown).

**Table 3:** Relationship between KCNMA1 amplification, proliferation, and expression of AR.

	score	KCNMA1	
		amplified	non-amplified
Ki67 LI*	low	2/9 (22%)	19/25 (76%)
	high	7/9 (78%)	6/25 (24%)
AR-IHC**	−	8/9 (89%)	11/25 (44%)
	+	1/9 (11%)	14/25 (56%)

* *Ki67 LI: scores range from 0 to 90, low scores <30, high scores ≥30.*

** *IHC: AR-status scored negative or positive.*

Percentage of tumours with high (≥30%) and low (<30%) Ki67 labelling index (LI). Amplification of KCNMA1 was associated with high Ki67 LI (p<0.002) and AR negative IHC status (p<0.05).

### Association between KCNMA1 amplification and BK mRNA expression

**Figure 5:**
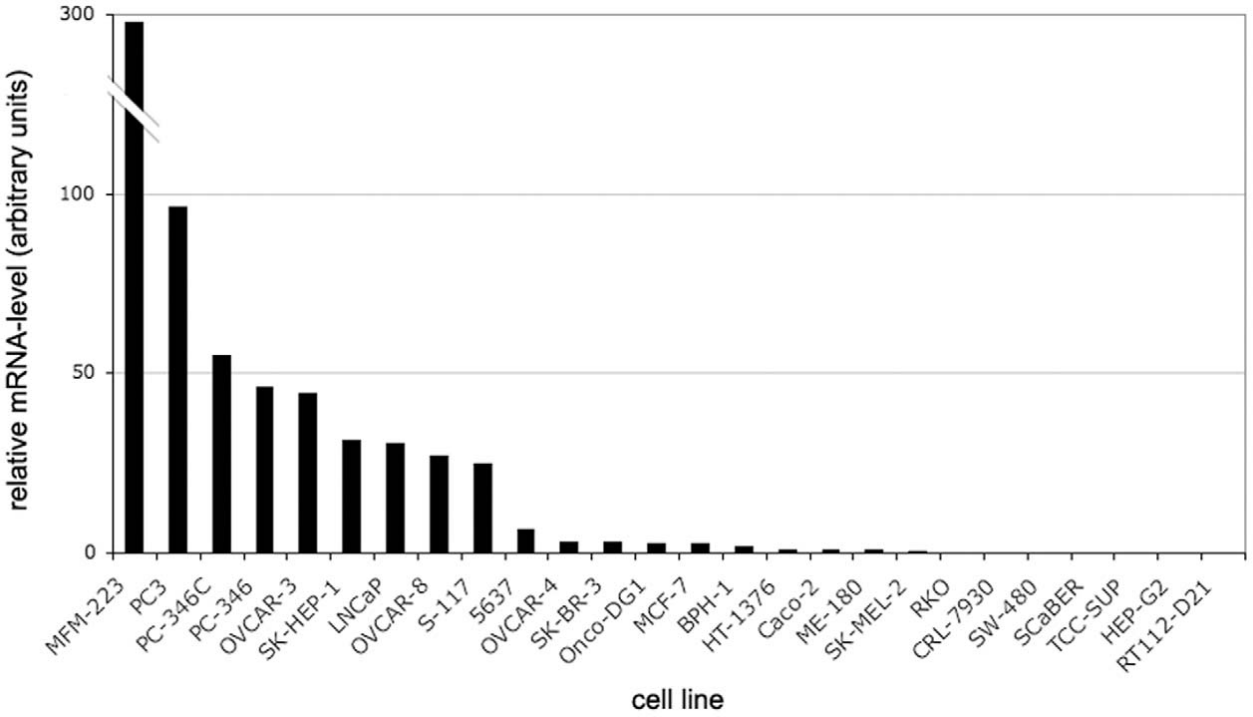
Amplification of KCNMA1 in cancer cell lines. Expression profiles of 26 cancer cell lines, derived from different tissue types (quantitative real-time PCR analysis). KCNMA1 amplified cell lines MFM223 and PC3 showed the highest expression.

We analyzed the expression profile of BK-mRNA using quantitative real-time PCR in 26 different cancer cell lines. All samples were normalized against GAPDH [33]. *KCNMA1* amplified breast (MFM223) and prostate (PC3) cell lines revealed highest expression of BK-mRNA, with transcript levels in MFM223 being over 100-times higher than in MCF7 (**Figure 5**). The T47D cell line harbors a deletion of GAPDH and was therefore excluded from further analysis. Since amplification of *KCNMA1* was restricted to gynecological and prostate cancers, we tested the association between BK-mRNA levels in cell lines originating from (a) hormone sensitive versus (b) hormone insensitive organs. The average rank of KCNMA1-mRNA expression was significantly higher in class (a) than in class (b) (19.1 versus 8.7; p<0.001; Wilcoxon signed-rank test) (**Table 4**).

**Table 4:** Cell lines.

Class	Cell line	Designation	Source	Tissue of origin
(a)	MFM-223	ACC-422	DSMZ	breast
(a)	SK-BR-3	HTB-30	ATCC	breast
(a)	MCF-7	HTB-22	NCI-60	breast
(a)	OVCAR-3	HTB-161	ATCC	ovary
(a)	OVCAR-8	-	NCI-60	ovary
(a)	OVCAR-4	-	NCI-60	ovary
(a)	Onco-DG1	ACC-507	DSMZ	ovary
(a)	PC3	CRL-1435	ATCC	prostate
(a)	PC-346C	-	Rotterdam	prostate
(a)	PC-346	-	Rotterdam	prostate
(a)	LNCaP	CRL-1740	ATCC	prostate
(a)	BPH-1	ACC 143	DSMZ	prostate
(b)	5637	HTB-9	DSMZ	bladder
(b)	HT-1376	CRL-1472	ATCC	bladder
(b)	CRL-7930	-	ATCC	bladder
(b)	SCaBER	HTB-3	ATCC	bladder
(b)	TCC-SUP	HTB-5	ATCC	bladder
(b)	RT112-D21	-	ATCC	bladder
(b)	ME-180	-	CTCC	cervix
(b)	Caco-2	CCL-185	ATCC	colon
(b)	RKO	CRL-2577	ATCC	colon
(b)	SW-480	CCL-228	ATCC	colon
(b)	SK-HEP-1	HTB-52	ATCC	liver
(b)	HEP-G2	HB-8065	ATCC	liver
(b)	SK-MEL-2	HTB-68	ATCC	skin
(b)	S-117	ACC-266	DSMZ	thyroid

Cell lines originating (a) with frequent and (b) without expression of sex hormone receptors.

### Contribution of BK channels to cell proliferation

**Figure 6:**
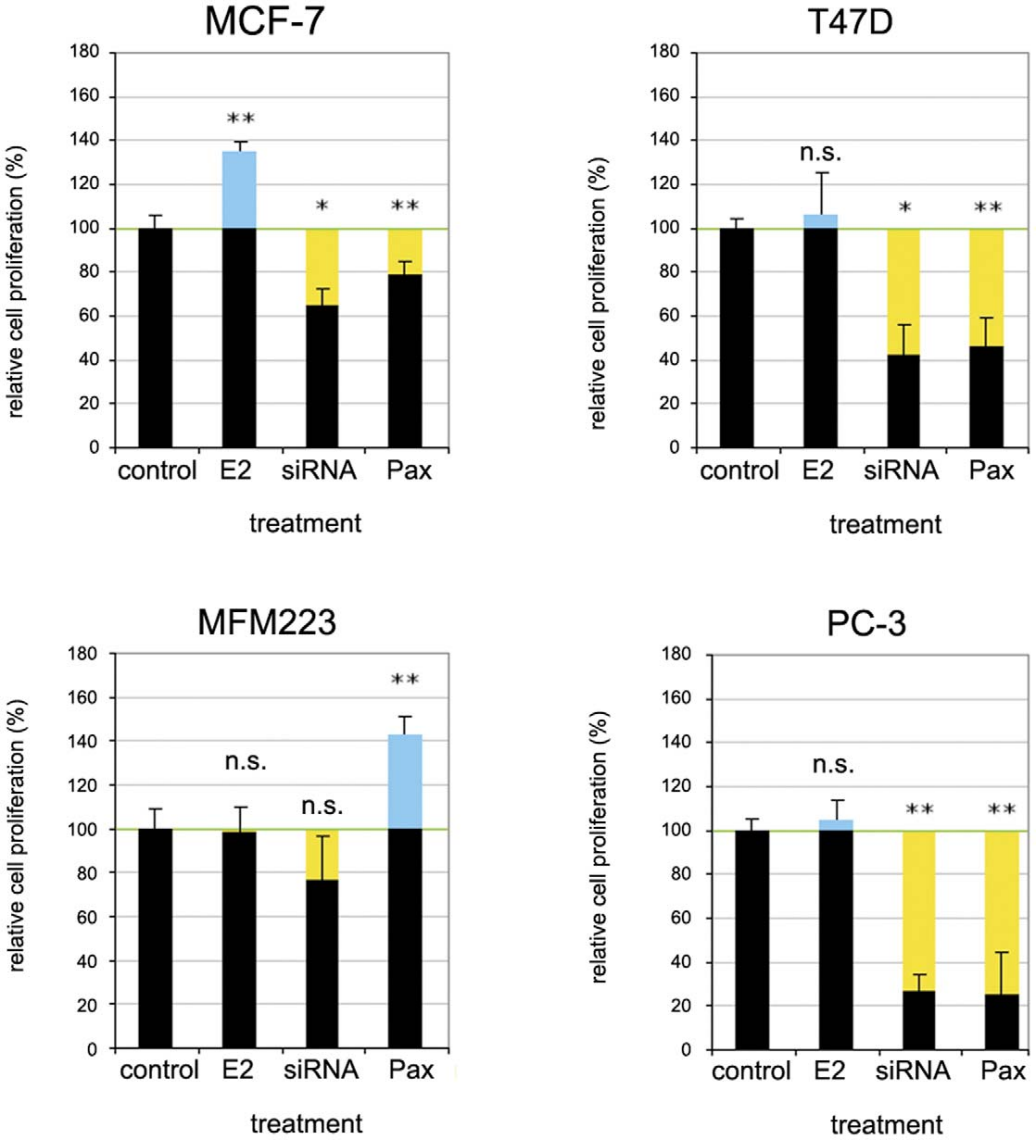
Proliferation of breast cancer cells. Proliferation rates of MCF7, T47D, MFM223 and PC3 in response to siRNA targeting KCNMA1, BK-channel blocker paxilline and 17*β*-estradiol (E2). Proliferation rates of MCF7 and T47D were significantly reduced by siRNA and paxilline. MFM223 did not show a similar reduction after RNAi and strongly increased the proliferation rate upon treatment with paxilline; * p<0.05, ** p<0.01, n.s. = not significant.

**Figure 7:**
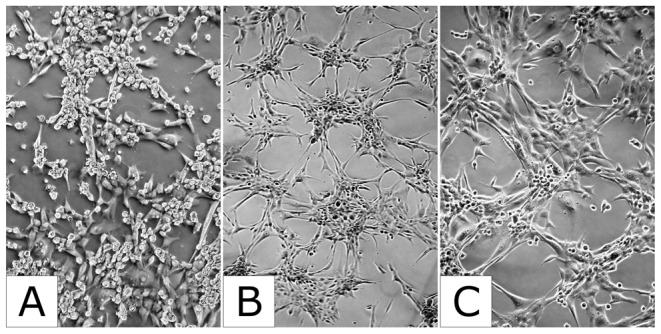
Effect paxilline on cell morphology. Cell shape of MFM223 cells before (A) and after the treatment with 15 µM KCNMA1 blocker paxilline (B and C). Images show cells at 20× (A, B) and 40× (C) magnification.

To investigate its functional role for cell proliferation, we knocked down KCNMA1 in the cell lines MFM223, MCF7 and T47D using KCNMA1-specific siRNA. Knocking down KCNMA1 reduced proliferation by 58% (T47D), 35% (MCF7), and 13% (MFM223) (**Figure 6**). The knockdown efficiencies were between 75–86% (measured after 24–48 h) across all analyzed cell lines (data not shown). Similarly, pharmacological inhibition of BK channels by paxilline decreased cell proliferation rate by 54% (T47D) and 21% (MCF7), but surprisingly enhanced proliferation by 42% in MFM223 cells, accompanied by a noticeable change in the cell morphology (**Figure 7**). In comparison, proliferation of the *KCNMA1*-amplified prostate cancer cell line PC3 was strongly reduced by both treatments (**Figure 6**). To test whether female sex hormone derivates increase the cell proliferation rate in these cell lines, we performed experiments in the presence of 17β-estradiol. 17β-estradiol is described to be an activator of BK channels and increased cell proliferation in MCF7, but not in T47D, MFM223 or PC3 cells (**Figure 6**).

### Functional analysis of BK channels in breast cancer cell lines

**Figure 8:**
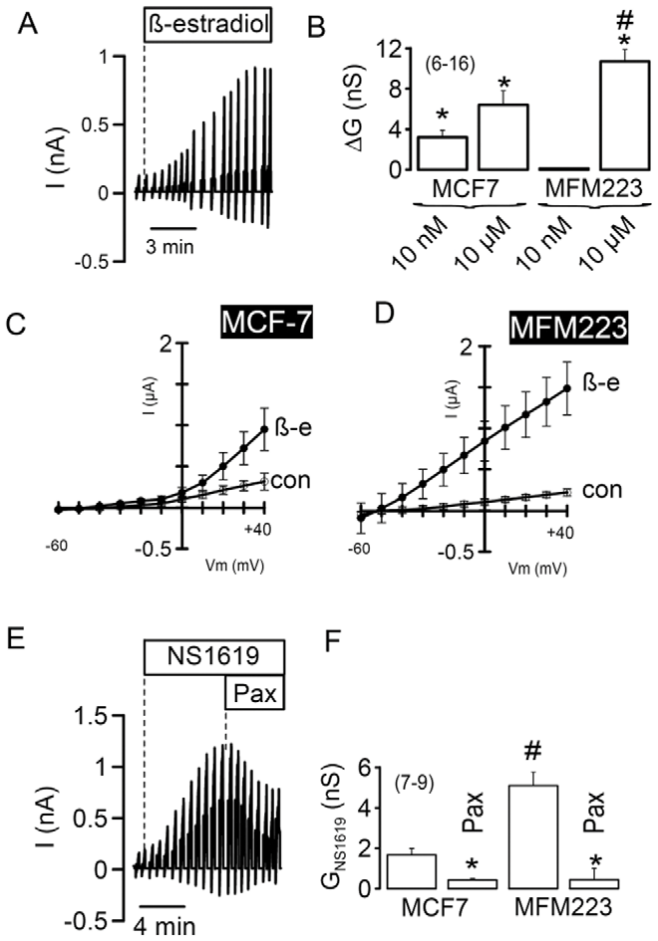
KCNMA1 currents in MCF7 and MFM223 cells. A) Continued whole cell current recording shows the activation of whole cell currents by 17*β*-estradiol (10 µM) in a MFM223 cell. B) Summary of the whole cell conductance activated in MCF7 and MFM223 cells by two different concentrations of 17*β*-estradiol (10 nM and 10 µM). C,D) Current/voltage relationships for MCF7 and MFM223 cells under control conditions and after stimulation with 17*β*-estradiol. E) Continued whole cell current recording shows the activation of whole cell currents by NS1619 (10 µM) in a MFM223 cell, and the inhibition of the NS1619-activated current by paxilline (Pax, 5 µM). F) Summary of the whole cell conductance activated by NS1619 in MCF7 and MFM223 cells and inhibition of the whole cell current by paxilline. Mean ± SEM, (n) = number of cells measured. *significant stimulation by 17*β*-estradiol or NS1619 (paired t-test). # significant difference when compared to MCF7 cells (unpaired t-test).

Electrophysiological analysis revealed that BK K^+^ currents were present in both MCF7 and MFM223 cell lines. Furthermore, we found that 17*β*-estradiol stimulated whole-cell K^+^ outward currents in both cell lines resulting in a hyperpolarisation of their membrane potential (**Figure 7 A,C**). While low (“physiological") concentration (10 nM) of 17*β*-estradiol was sufficient to activate BK K^+^ currents in MCF7 cells, stimulation of KCNMA1-driven K^+^ currents in MFM223 cells required a 1000-fold higher (“non-physiological") concentration (10 µM). This finding suggests a different sex hormone-sensitivity towards KCNMA1 stimulation in these cell lines (**Figure 8 B–D**). A direct activator of KCNMA1, NS1619 (10 µM), clearly activated BK currents in MFM223 cells, but had little effects in MCF7 cells (**Figure 8 E,F**). K^+^ outward currents activated by 17*β*-estradiol or NS1619 were inhibited by the BK channel blocker paxilline (5 µM). Taken together, these results indicate that breast cancer cells exhibit functional BK currents. Fast proliferating MFM223 cells have larger KCNMA1 currents with pharmacological properties different to MCF7 cells, possibly due to differential expression of ß-subunits (KCNMB1-4).

**Figure 9:**
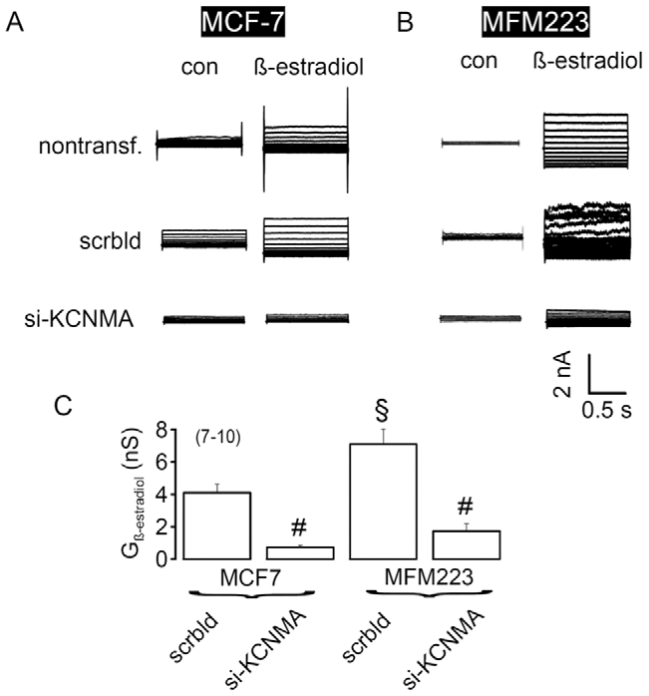
Suppression of KCNMA1 in MCF7 and MFM223 cells by siRNA. A) Whole cell currents in non-transfected MCF7 cells and cells transfected with nsRNAi (control) and siRNA targeting KCNMA1. B) Whole cell currents in: non-transfected MFM223 cells and cells transfected with nsRNAi and siRNA targeting KCNMA1. C) Summary of the whole cell conductance activated by 17*β*-estradiol (10 µM) in MCF7 and MFM223 cells and inhibition of the whole cell currents by siRNA against KCNMA1. Mean ± SEM, (n) = number of cells measured. ^§^significant difference when compared to MCF7 cells (unpaired t-test). # significant difference when compared to cells treated with scrambled RNAi (unpaired t-test).

To verify the above presented results based on the use of pharmacological tools, RNA-interference was performed using siRNA against *KCNMA1* to suppress KCNMA1-currents in both MCF7 and MFM223 cells. While 17*β*-estradiol clearly activated whole-cell K^+^ outward currents in non-treated MCF7 (10 nM) and MFM223 (10 µM) cells and in cells transfected with scrambled RNA, K^+^ current activation by 17*β*-estradiol was strongly reduced in both cell lines after siRNA-knockdown of KCNMA1 (**Figure 9 C**).

## Discussion

Our study reveals for the first time that *KCNMA1* amplification is restricted to human cancer types that derive from tissues regulated by sex steroid hormones including prostate, breast, uterus, and ovary. In breast cancer, *KCNMA1* is amplified in a rare subgroup of breast cancers with poor prognosis. Moreover, our data suggest that 17*β*-estradiol can induce proliferation of breast cancer cells through activation of BK channel.

K^+^ channels control cellular functions that determine tumour malignancy such as cell cycle and survival, proliferation, and migration. Previous studies have firmly established that different types of K^+^ channels exhibit oncogenic potential [10], [11], [12], [13], [14], [15], [16]. Large conductance, voltage- and Ca^2+^-activated potassium channel (KCNMA1; BK) is located at 10q22.3 and was recently found to be amplified in a proportion of castration-refractory human prostate cancers, suggesting an involvement in the progression of the disease [13]. In the present study, we investigated the prevalence of the *KCNMA1* amplification beyond prostate cancer. Our findings provide the first evidence that breast, ovarian and endometrial cancers carry also *KCNMA1* amplification. Highest prevalence was found in invasive ductal breast cancer and serous carcinoma of the ovary and endometrium (3–7%). Our results demonstrate that *KCNMA1* amplification is associated with high tumour stage, high tumour grade, high tumour cell proliferation, and poor prognosis.

*KCNMA1* was amplified in MFM223, a rapidly growing metastatic human breast cancer cell line, and associated with strongly up-regulated mRNA- and protein expression. KCNMA1-mRNA expression was by far highest in MFM223 among all 26 analyzed cancer cell lines using quantitative real-time PCR, and more than 100-fold higher than in the non-amplified breast cancer cell line MCF7. Thus, it appears paradoxical that there was no inhibition of proliferation by siRNA or paxilline in MFM223 as opposed to the breast cancer cell lines MCF7 and T47D. One could hypothesize that the massive overexpression renders the MFM223 cells resistant to inhibition. Moreover this cell line essentially grows estrogen independent and demonstrates pharmacological properties different to other BK-expressing cancer cells, which is possibly due to differential expression of ß-subunits. In contrast to MCF7 and T47D, MFM223 has a low ER and PR expression but a very strong AR expression, [27], [34]. This indicates that the sex hormone receptor status might also affect the response of breast cancer cells to inhibition of BK channel.

Although expression and function of KCNMA1 is related to cell cycle control and correlates with proliferation and migration, it is not a simple task to unravel the underlying mechanisms [10]. BK channels interact with a large number of proteins, regulatory factors and second messenger pathways, which makes it difficult to predict a general role for KCNMA1 in different tissues. Moreover experimental conditions necessarily differ between the methods used to examine the role of channel expression and function for cell proliferation, e.g. patch clamp experiments are done in the absence of serum [10].

Along this line, in the non-estrogen sensitive MFM223 cells, BK currents were activated only by non-physiologically high concentrations of 17*β*-estradiol, while low concentrations (10 nM) were sufficient to activate BK currents in MCF7 cells. A direct activator of KCNMA1, NS1619 (10 µM), significantly activated BK currents in MFK223 but not in MCF7 cells. These results suggest that, apart from differences in the level of KCNMA1 expression, there are probably differential expression levels of KCNMB1-4 subunits in both cell lines, which may lead to differential sensitivity towards physiological agonists (17*β*-estradiol) and pharmacological compounds (NS1619). Alternative pre-mRNA splicing is another mechanism that could explain different function of BK channels in different tissues, tumour types and cellular conditions [35]. For example, it has been shown that estrogen can influence the response of KCNMA1 to protein kinase A by differentially regulating the expression of a splice variant in rat myometrium [36].

The restriction of *KCNMA1* amplification to carcinomas from sex hormone-regulated organs is highly intriguing and also suggests an interaction of KCNMA1 within the hormonal context of these tumours. This is supported by our data, showing that 17*β*-estradiol induces cell proliferation in MCF7 and functional activation of BK channel in MCF7 and MFM223, which is abrogated by siRNA or by paxilline. This is also in line with a previous study where the BK blocker iberiotoxin lead to reduced proliferation in the breast cancer cell line MDA-MB-231 [37]. In addition, it has been shown that BK channel activity in MCF7 can be stimulated by tamoxifen, a therapeutic ER antagonist with partial agonistic activity, leading to increased cellular proliferation [21]. In contrast, others were unable to confirm an enhanced proliferation through activation of BK channels in MCF7 [23]. Notably, one report showed a role for KCNMA1 in breast cancer invasion and metastasis to brain [38].

We found an inverse association between *KCNMA1* amplification and AR expression. Almost all (8 of 9; 89%) *KCNMA1* amplified, but only 11 of 25 (44%) non-amplified breast cancers were AR-negative. The role of AR in breast cancer has not yet been fully elucidated, but it has been postulated that AR has an anti-proliferative and favourable prognostic effect in ER-positive breast cancer, whereas it has a growth-stimulating and unfavourable prognostic effect in ER-negative breast cancer [39].

It has previously been demonstrated that not only 17*β*-estradiol but also testosterone can relax smooth muscle by potentiating BK channels [40]. Responsiveness to these sex hormones could explain why BK channel up-regulation and *KCNMA1* amplification occur in hormone-regulated cancers. Further studies are needed to elucidate the complex interplay between sex hormones and KCNMA1 in breast- and prostate cancer that is likely to be influenced by the type of regulatory ß-subunits of the BK channel, alternative splicing, the background of ER and/or AR status, and anti-hormone therapy.

Unfortunately, there is currently no reliable antibody for formalin-fixed and paraffin-embedded material. Therefore, we could not test the prevalence and significance of KCNMA1 protein expression in a large number of clinical cancer specimens. However, immunofluorescence revealed moderate to strong KCNMA1 expression in eight out of nine frozen breast cancer specimens irrespectively of *KCNMA1* gene copy number. Thus, the presence of the *KCNMA1* amplification in tumour specimens and MFM223 might rather highlight *KCNMA1* as a gene with oncogenic relevance, while mechanisms other than amplification seem to be responsible for KCNMA1 overexpression in most of these tumours.

In conclusion, we report for the first time that *KCNMA1* amplification occurs in carcinomas of the breast, ovary and endometrium. The prevalence of the amplification across different human cancer types points towards a broader general oncogenic potential of KCNMA1 than previously assumed.
